# Modeling Maturity Onset Diabetes of the Young in Pluripotent Stem Cells: Challenges and Achievements

**DOI:** 10.3389/fendo.2021.622940

**Published:** 2021-02-22

**Authors:** Carmel Braverman-Gross, Nissim Benvenisty

**Affiliations:** The Azrieli Center for Stem Cells and Genetic Research, Department of Genetics, The Alexander Silberman Institute of Life Sciences, The Hebrew University of Jerusalem, Jerusalem, Israel

**Keywords:** maturity onset diabetes of the young, human embryonic stem cells, induced pluripotent stem cells, disease modeling, genetic and epigenetic aberrations

## Abstract

Maturity onset diabetes of the young (MODY), is a group of monogenic diabetes disorders. Rodent models for MODY do not fully recapitulate the human phenotypes, calling for models generated in human cells. Human pluripotent stem cells (hPSCs), capable of differentiation towards pancreatic cells, possess a great opportunity to model MODY disorders *in vitro*. Here, we review the models for MODY diseases in hPSCs to date and the molecular lessons learnt from them. We also discuss the limitations and challenges that these types of models are still facing.

## Introduction

Monogenic diabetes refers to a group of disorders caused by mutations in a single gene resulting in diabetes. To date, more than 40 subtypes of monogenic diabetes have been identified, most of them results in *β* cell loss or function impairment. In rare cases, diabetes is caused by mutations leading to insulin resistance or associated with other features of genetic syndromes affecting multiple organs. The typical classification of monogenic diabetes includes two main subgroups; neonatal diabetes, usually presenting before 6 months of age, and maturity onset diabetes of the young (MODY), usually presenting in youth and adults. While neonatal diabetic cases are rare (1 in 1,000,000 birth), MODY disorders are more common, accounting for 1–5% of all diabetic cases (1–4). The acronym MODY was first used in 1975 by Fajans and Tattersall, to distinguish a hereditary form of diabetes presented in juvenile patients from classical type 1 diabetes patients (5). Today, the term MODY is used to describe a group of clinically heterogeneous metabolic disorders that are characterized by pancreatic *β* cell functional impairment. The clinical features of MODY are varied and depend on the causal gene. Some of the common features of MODY include hyperglycemia, diagnosed usually in childhood or adolescence (under 25), family history (autosomal dominant inheritance) and lack of pancreatic auto-antibodies (2, 6).

## Maturity Onset Diabetes of the Young Genetics and Pathogenesis

Fourteen distinct subtypes of MODY have been identified to date, all caused by mutations in genes important for pancreatic *β* cell development, regulation, and function. Most of these genes encode for transcription factors (TFs), *i.e.*, MODY1, MODY3, MODY4, MODY5, MODY6, MODY7, and MODY9 (caused by mutations in the genes *HNF4A*, *HNF1A*, *PDX1*, *HNF1B*, *NEOROD1*, *KLF11*, and *PAX4*, respectively). Some of them encode for enzymes, *i.e.*, MODY2, MODY8, and MODY11 (caused by mutation in the genes *GCK*, *CEL*, and *BLK*, respectively), and some involve other pancreatic genes, *i.e.*, MODY10, MODY12, MODY13 and MODY14 (caused by mutations in *INS*, *SUR1*, *KCNJ11*, and *APPL1* genes, respectively) (7–9).

Clinical diagnosis of MODY is still suboptimal, mainly due to the variability of clinical presentations and their similarity to symptoms of other types of diabetes, leading to misdiagnosis of MODY as type 1 or type 2 diabetes (10, 11). However, with the increasing availability and price reduction of genetic tests, MODY diagnosis is rising. An accurate and timely diagnosis of MODY can dramatically affect the medical treatment given as treatment is tailored to the specific mutation. This treatment is often dramatically different from that of type 1 or type 2 diabetes (12). Precise diagnosis is also important for early identification of asymptomatic or undiagnosed family members, in order to minimize the disorder’s impact on multiple organs (1).

Some of the MODY genes are specifically involved in *β* cells’ function, while others are related to different stages of the endocrine pancreatic development. Studying the effect of perturbation in these latter genes may also help understand the developmental processes and pathogenesis of other pancreatic diseases. Furthermore, understanding the mechanisms underlying *β* cell formation could improve *in vitro* differentiation protocols of *β* cells from human stem cells, enhancing the feasibility of pancreatic islet transplantation in type 1 diabetes patients and other pancreatic disorders.

## Modeling Maturity Onset Diabetes of the Young

**Figure 1:**
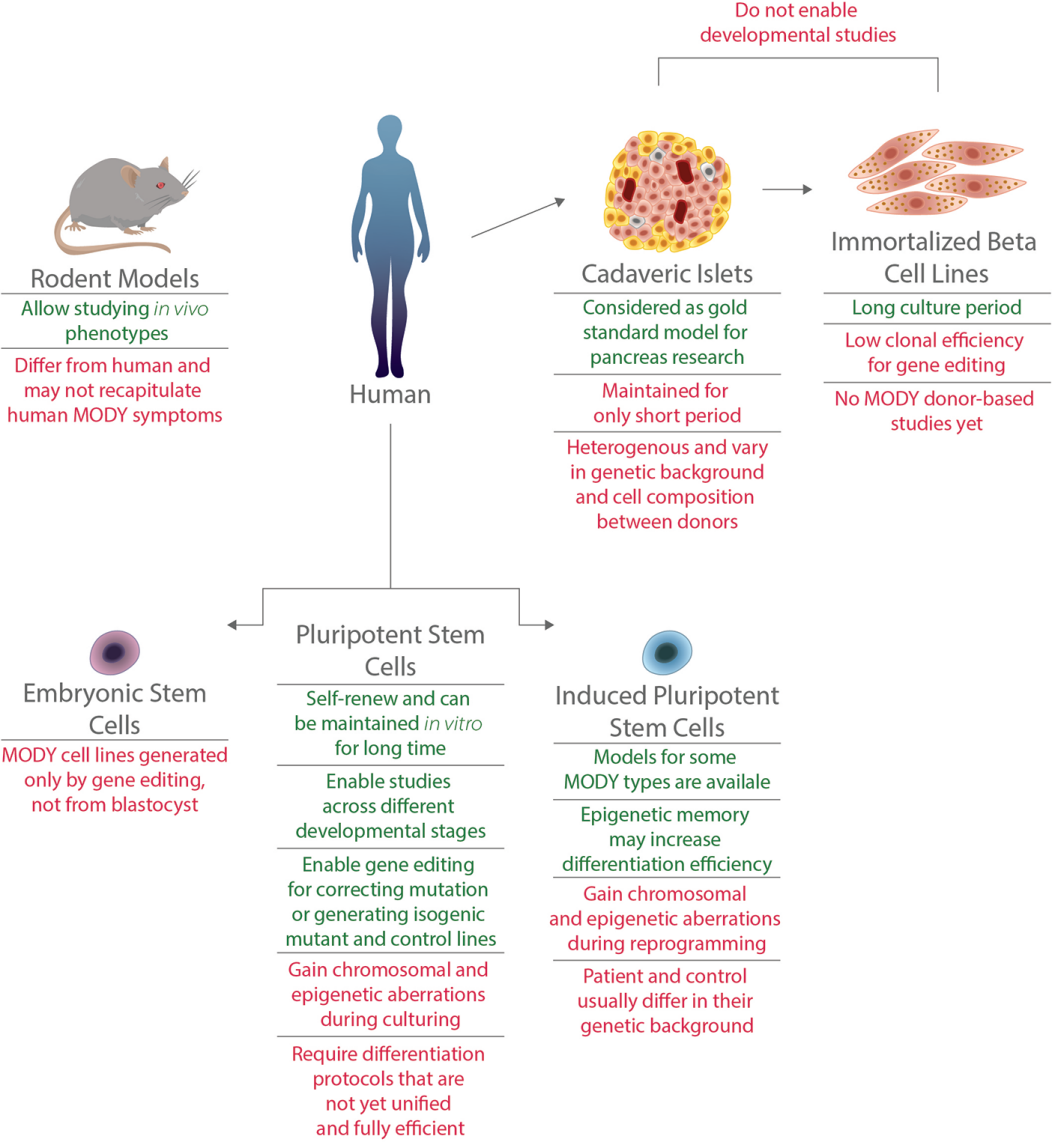
Advantages and limitations of various models for MODY disorders. Models for MODY disorders can be generated using different *in vivo* and *in vitro* systems, such as rodents, human cadaveric islets, immortalized beta cell lines, hiPSCs and hESCs. Advantages (appear in green) and disadvantages (appear in red) for each type of model are presented.

A great part of the current understanding of pancreatic *β* cell development and function was achieved using rodent models. However, as pancreatic development and architecture, as well as glucose response, differ substantially between mice and humans, rodent models do not always accurately represent the human phenotypes. Such are the cases of heterozygous mutations in the genes *ABCC8*, *HNF1B*, *HNF4A*, *HNF1A*, *GATA4*, and *GATA6* that cause neonatal diabetes or MODY in humans, but do not present any diabetic pathophysiology in mice (13–15) (**Figure 1**).

Human studies of diabetes mechanisms can also be done by using cadaveric islets, which are human primary islets harvested *post mortem* from pancreatic donors (15, 16),. Recent studies that used human islets from donors diagnosed with type 1 diabetes revealed mutations in genes causing monogenic diabetes, including MODY, that are the primary cause of diabetic symptoms (17, 18). This approach is limited due to high variability between islets, the short life span of the cells composing them and mainly due to low donor accessibility. The latter is especially challenging when modeling MODY, given the low prevalence and diagnosis of this disease (17, 18). Immortalized *β* cells, or *β* cell lines, are another human cell type that are used in the field of diabetes research. To date, no cell lines were established from MODY patients, calling for gene editing to model these diseases. Although CRISPR/Cas9 editing has been previously used in EndoC-BH lines (19), these lines have low clonal efficiency which makes editing at clonal level challenging (15, 20). In addition, both cadaveric islets and immortalized *β* cells enable the study of mature pancreatic cells and are less suitable for studying genes that have a role during pancreatic development (**Figure 1**).

This calls for suitable monogenic diabetes models that can be fulfilled by human pluripotent stem cells (hPSCs). Both induced pluripotent stem cells (iPSCs) and embryonic stem cells (ESCs) can be differentiated from their pluripotent state to pancreatic *β*-like cells. Over the last decade, such differentiation protocols were developed and improved, enabling the generation of functional human *β* cells (21–25). Glucose-responsive and insulin secreting *β* cells can be generated *in vitro*, and were even proved to reduce blood glucose levels when transplanted in Streptozotocin-induced diabetic mice. Differentiation protocols are performed in a stepwise manner, typically mimicking *in vivo* development of *β* cells (26). By using PSC-derived cells carrying a mutation in MODY-causing gene, one can follow the natural course of development and elucidate the relevant stage or stages that are interrupted by the mutation (**Figure 1**).

Since MODY genes vary in their function and in the developmental stages in which they are expressed and act, there may be different mechanisms underlying each of the MODY disorders. These mechanisms can be studied using PSCs.

## iPSC Models for Maturity Onset Diabetes of the Young

hPSC models for several MODY types were established in the past decade (**Table 1**), mainly for the manifestations caused by mutant transcription factors. The vast majority of these models were based on iPSCs. iPSCs are generated by reprogramming patients’ somatic cells, harboring the mutated gene. These reprogrammed cells are capable of self-renewal and can be maintained *in vitro*. They can be further differentiated into pancreatic cells, enabling the study of the effects of particular mutations.

**Table 1:** MODY models in hPSCs.

Type	Gene Name	Mutation Type	Type of cells	Reference
MODY1	HNF4A	p.I271fs	hiPSC	(27)
		p.I271fs	hiPSC	(28)
		p.I271fs	hiPSC	(29)
		p.Q268X	hiPSC	(30)
MODY2	GCK	V62A	hiPSC	(27)
MODY3	HNF1A	p.P291fsinsC	hiPSC	(27)
		p.R271W and p.P379fs	hiPSC	(31)
		p.S142F	hiPSC	(32)
		19/16bp δ-exon 1 indel-exon 1	hESC human *β* cell lines	(19) (19)
		p.P291fsinsC	hiPSC	(33)
MODY4	PDX1	p.P33T	hiPSC	(34)
		p.C18R	hiPSC	(35)
		p.L36fs and p.A34fs	hESC	(36)
MODY5	HNF1B	g.1-1671del	hiPSC	(27)
		p.R177X	hiPSC	(37)
		p.S148L	hiPSC	(38)
MODY8	CEL	p.C563fsX673	hiPSC	(27)
MODY13	KCNJ11	p.E227K	hiPSC	(39)

Such models were generated for MODY1 (27–30), MODY2 (27), MODY3 (27, 31–33), MODY4 (34, 35), MODY5 (27, 37, 38), MODY8 (27), and MODY13 (39) (**Table 1**).

These studies modeled different mutations and used different differentiation protocols, leading to complex conclusions in some cases. Nevertheless, these studies contribute to our understanding of the relations between TFs and their gene targets throughout pancreatic development and of the mechanisms underlying MODY symptoms. We will therefore focus on the analyses of MODY disorders that involve TFs.

### Maturity Onset Diabetes of the Young 1

MODY1 is characterized by progressive *β* cell dysfunction, macrosomia, and neonatal hyperinsulinemic hypoglycemia in some of the cases. In 2017, Vethe et al. generated iPSCs from patients carrying I271fs mutation in *HNF4A* gene and differentiated them towards *β*-like cells (28). Comparing MODY1 iPSCs to iPSCs from a healthy member of the same family, they reported that the mutation did not alter the differentiation capacity of the cells. Mutated and control cells expressed similar levels of insulin, as well as other pancreatic proteins such as PDX1, NEUROD1 and MAFB. Comparing the proteomic landscape and function (by glucose stimulated insulin secretion (GSIS) test) of mutant and control *β*-like cells to adult human islet, they reported an immature state of the differentiated iPSCs.

A recent study from the same group by Ghila et al. used the same MODY1 iPSCs differentiated towards hormone producing islet-like cells, to investigate their miRNA expression profile (40). Their bioinformatic analysis revealed miRNAs that are differentially expressed between mutant and control cells. These miRNAs are composed of two subgroups, one that showed different expression during early differentiation stages (during the formation of posterior foregut) and the other was differentially expressed in later stages (after pancreatic endoderm formation). They observed alterations in miRNA networks related to *TP53* regulation in both stages. A combination of the miRNA data with transcriptomic data in MODY1 cells further highlighted an activation of *TP53* in the MODY1 cells, suggesting that mutations in *HNF4A* affect cell cycle arrest during late differentiation.

Ng et al. used iPSCs generated from a MODY1 patient carrying the same I271fs mutation. They showed that this mutation has haploinsufficiency, rather than a dominant negative mode of action, and that it leads to cytoplasmic mislocalization of the HNF4A TF (29). In order to assess the mutation effect on the developmental process, they focused on the hepatopancreatic foregut endoderm stage, an early stage of pancreatic differentiation. RNA sequencing (RNAseq) data of MODY1 cells showed an upregulation of hindgut HOX genes in parallel to downregulation of a set of pancreatic and liver genes including *GATA4*, *HNF1B*, and *PDX1*. Together, these results suggested that HNF4A has a role in hindgut repression. Downregulation of hepatic genes was also detected in further differentiated cells towards hepatocytes, while a significant reduction in *HNF1A* was detected in differentiated pancreatic *β* cells.

Braverman-Gross et al. generated MODY1 iPSCs from patients carrying a Q268X mutation in the *HNF4A* gene [previously shown to cause haploinsufficiency (41),]. The authors differentiated the cells towards pancreatic progenitors and exhibited an upregulation of some pancreatic markers, such as *PAX6*, *NEUROD1* and *NEOUROG3* in the MODY1 cells, indicating a possible partial compensatory mechanism for the mutation (30). RNAseq data of MODY1 cells from the primitive gut tube stage of differentiation revealed subgroups of putative HNF4A gene targets. Targets with less HNF4A binding sites and more sites for other TFs in their promotors were less affected by HNF4A haploinsufficiency, further suggesting a redundancy mechanism for the mutation.

### Maturity Onset Diabetes of the Young 3

MODY3, caused by mutations in another TF, HNF1A, is characterized by gradual *β* cell dysfunction and progressive hyperglycemia. In 2015, Stepniewsky et al. generated two MODY3 iPSC lines derived from patients carrying either R271W or P379fs mutations in *HNF1A* (31). They then differentiated the cells towards hormone producing cells. Even though the differentiation capacity was rather poor and resulted in immature polyhormonal cells with low percentage of insulin-positive cells, they showed that the MODY3 cells had a similar capacity to differentiate as iPSCs generated from healthy individuals (31).

Yabe et al. generated MODY3 iPSCs carrying the most common mutation in *HNF1A* gene, P291fsinsC (33). They differentiated the iPSCs to pancreatic cells, and showed that the mutated RNA is abolished by nonsense mediate decay (NMD), consequently leading to minimal expression of the mutant protein, indicating haploinsufficiency mechanism for this MODY3 mutation as well.

### Maturity Onset Diabetes of the Young 4

MODY4 is caused by mutated PDX1 TF, which usually results in defective insulin secretion, while homozygous mutations in this gene also lead to pancreatic agenesis. In 2016, Wang et al. generated two iPSC lines carrying heterozygous P33T and C18R mutations in *PDX1* gene (34, 35). By differentiating the cells towards pancreatic endoderm, they exhibited no difference in PDX1 expression and early pancreatic differentiation between mutant and control cells (42). At later stages, *β*-like cells derived from MODY4 iPSCs had low insulin expression and reduced GSIS. Using CRISPR/Cas9 gene editing, they also induced the same mutations in homozygous state, as well as heterozygous frame shift mutation in control iPSCs. The mutated cells showed impairment in differentiation towards pancreatic precursors by reduced C-peptide expression and reduced GSIS. In addition, they showed reduction of transcription levels in pancreatic genes including *NEUROD1*, *ISL1*, and *INS*, which were previously shown to be direct PDX1 targets (43).

### Maturity Onset Diabetes of the Young 5

MODY5, caused by mutations in HNF1B TF, is characterized by reduced insulin secretion and renal cysts in some patients. Yabe et al. generated MODY5 iPSCs from a patient carrying R177X mutation in *HNF1B* (37). As in the case of their MODY3 study, they showed that NMD destruction of the mutant RNA is taking place, hinting for haploinsufficiency of HNF1B as the underlying cause of MODY5. Teo et al. generated iPSCs from MODY5 patients, carrying a different mutation, S148L (38). Differentiated MODY5 cells showed upregulation of definitive endoderm and early pancreatic genes, including *SOX17*, *FOXA2*, *GATA4*, *GATA6* and *PDX1*, as well as *HNF1B* itself, suggesting a compensatory gene expression circuit. Using luciferase assays they also showed that the increase in *PDX1* expression was directly related to the S148L mutant allele. The only pancreatic gene that was downregulated in the MODY5 cells was *PAX6*. It was hypothesized that this occurred by an indirect regulation, as *PAX6* promoter was not found to bind HNF1B. This downregulation could explain some of the diabetic symptoms of MODY5 patients.

## Embryonic Stem Cell Models for Maturity Onset Diabetes of the Young

The scarcity of MODY patients and tissues donors, as well as inherent disadvantages of iPSC-based disease models, as discussed below, call for additional *in vitro* MODY models. One of the promising models currently being used are based on targeted gene editing, which became much simpler and prevalent with the introduction of the CRISPR/Cas9 methodology. Some groups recently combined gene editing of human embryonic stem cells together with pancreatic differentiation, to study the effects of MODY-related gene disruption (**Table 1**).

In 2016, Zhu et al. used the HUES8 ESC line to generate 62 ESC sublines carrying mutations in eight different pancreatic TFs, in order to study their role in poly hormonal *β*-like cell differentiation. One of these genes was *PDX1*, the gene causing MODY4 disease, for which they created two homozygous lines carrying biallelic frame shift mutation, L36fs and A34fs (36). ESCs carrying the same heterozygous monoallelic mutations showed a reduction in PDX1 expression compared to WT cells, indicating haploinsufficiency mechanism for these mutants, leading to decrease in endocrine hormone gene expression (*INS*, *SST*, *GCG*, *GHRL*) in differentiated cells.

Cardenas-Diaz et al. generated HNF1A homozygous and heterozygous deletion mutations in MEL1 and H1 ESC lines (19). They differentiated the cells and showed effect of the mutation in the *β*-like cells’ stage, where they observed a reduction in the expression of pancreatic TFs including *PDX1*, *RFX6*, *HNF4A* and *PAX4*. Mutant cells showed an increase in *ARX* gene levels, accompanied by higher GCG levels and lower *INS* expression in the mutant cells. These results indicated a role for *HNF1*A in inhibiting *α* cell development. Mutations in *HNF1A* also impaired the *β*-like cells GSIS, and cell with heterozygous mutations showed decreased mitochondrial respiration. In addition, they revealed a downregulation in the heterozygous cells of *LINKA*, a primate-specific lncRNA, and suggested it has a role as a mediator of *HNF1A* regulation for subset of *HNF1A* targets, specifically related to mitochondrial respiration and pancreatic genes expression.

## Lessons Learned From Maturity Onset Diabetes of the Young Models in PSCs

The diverse studies discussed above shed light on roles of TFs during the different stages of pancreatic development. Some of these TFs may have roles in a specific time point of the differentiation. Focusing on that exact step may be crucial to understanding the effects of a gene mutation. A mutation in *PDX1*, for example, was shown to affect the cells only at the terminal stage of differentiation (42). In contrast, mutations in *HNF4A* were found to alter foregut formation (29) and affect posterior foregut genes (30). Although insulin expression was reduced in mutant *HNF4A* pancreatic *β*-like cells in one study (29), in another, similar cells did not functionally differ from control cells (28). It is possible that compensatory mechanisms overcome the mutation and enable the mutant cells to fully or partially differentiate towards *β*-like cells. Such compensatory mechanism was shown for cells carrying mutations in *HNF1B*, where the mutant protein caused a possible direct upregulation of *HNF1B* itself and of *HLXB9*, and indirect upregulation of *PDX1* and other early pancreatic genes (38). HNF genes, including *HNF4A*, *HNF1A* and *HNF1B* are known to cross-regulate each other during pancreas development (44), and may cause the upregulation of shared targets when one of them is mutated. It is also likely that the variability of differentiation protocols used in different studies influence the final outcome (45)—highlighting the need for a uniform, efficient differentiation protocol, from the pluripotent stage to *β* cells.

Within each of the 14 known genes causing MODY, various mutations have been identified. The vast majority of MODY mutations are heterozygous, however, in some of the MODY genes a biallelic mutation may occur, usually leading to a more severe phenotype. Homozygous mutations in GCG, PDX1, ABCC8, and KCNJ11 cause permanent or transient neonatal diabetes, as well as pancreatic agenesis (as is the case in PDX1) or neurological abnormalities (in NEUROD1) (6, 9). MODY models in iPSCs were only generated from heterozygous patients, but gene editing using CRISPR/Cas9 studies generated homozygous cells that showed some differences from their heterozygous parallels. For example, cells homozygous for the P33T mutation in *PDX1* gene showed impairment in pancreatic differentiation to a much higher extent than the heterozygous cells (42). HNF1A homozygous mutants showed lower insulin levels than the heterozygous upon differentiation while heterozygous mutation affected the cellular respiration more than the homozygous knock-out mutation (19).

Since MODY disorders are typically characterized by heterozygous mutations, and symptoms of patients with homozygous mutations are distinct, it is hypothesized that a dosage dependent mechanism underlies MODY genes’ mode of action. One such dosage dependent model is haploinsufficiency (29, 33, 36, 37, 41). Thus, in a patient with heterozygous mutation, only one allele forms the gene product which is not enough for the normal function of the protein. The mechanisms underlying haploinsufficiency are varied and include threshold requirement of protein amount (*i.e.* in the case of TF that has to bind multiple sites of the DNA), or imbalanced stoichiometry of multi subunits complexes (46). Understanding the exact mechanism of action is important for future therapeutic purposes. While some haploinsufficiency genes are dosage sensitive and their upregulation may be toxic (46, 47), in other cases, activation of the normal allele transcription may rescue the haploinsufficiency disease symptoms (48).

MODY symptoms are affected not only from the mono or biallelic expression of a mutation, but also from the specific type of mutation, even within the same gene. Hence, specific mutations in a gene can cause MODY, while other mutations in the same gene can cause other types of disease. Permanent neonatal diabetes can be caused by mutations in *INS* and *KCNJ11* genes, while other mutations in these genes will cause MODY10 and MODY13, respectively, with symptoms presenting only later in life; congenital hyperinsulinism can be caused by mutations in *ABCC8*, *KCNJ11*, *GCK*, *HNF1A*, and *HNF4A* genes, the same genes that, when harboring different mutations, cause MODY12, MODY13, MODY2, MODY3, and MODY1, respectively (9, 49, 50). Even within patients with the same type of MODY, symptoms and cellular gene profiling may be different due to different types of mutations. For example, cells carrying P33T mutation in *PDX1* show lower levels of PDX1, NKX6.1 and C-peptide than cells carrying C18R mutation in the same gene (42).

Although generating a specific heterozygous mutation may be technically challenging (51), these observations highlight the need to model the exact type and location of a mutation, especially when using gene editing.

## Challenges of PSC Based Maturity Onset Diabetes of the Young Modeling

Using hPSCs for disease modeling, specifically monogenic developmental diseases such as MODY, have great benefits (52). In the case of MODY, one of the most important reasons to use hPSCs is the lack of appropriate animal models that recapitulate the human diabetic phenotypes (13). Furthermore, using hPSCs enables studying the complex pancreatic developmental process and assessing the effects of different mutations on its progress. This cannot be achieved using adult, differentiated tissues. However, when modeling a disease using hPSC, there are several issues one should consider (**Figure 1**).

### Genomic Aberrations

Since hPSCs are cultured and passaged *in vitro*, and iPSCs are going through the clonal process of reprogramming; they are prone to gain genomic aberrations. Such aberrations include large chromosomal aberrations, sub-chromosomal aberrations, copy number variations (CNVs), and point mutations (53, 54). These changes in genome content may affect the differentiation performance and global gene expression (55).

Although a normal karyotype is validated in all MODY iPSC models, usually by Giemsa band staining, in order to avoid clones containing large chromosomal gains or deletions, smaller mutations are more difficult to identify. Of note, it was found that hPSCs bear CNV hot spots, mainly on chromosomes 1, 12, 17q, 20q, and X (56). This finding may be crucial when modeling genes located within these genomic loci. These genes include *HNF4A*, located on chromosome 20q13, *HNF1A* on chromosome 12q24, and *HNF1B* on chromosome 17q12.

Recent studies showed that some hPSC lines harbor point mutations mainly in *TP53*, coding for the tumor suppressor p53 (57). p53 is a master regulator of apoptosis, cell cycle, and proliferation and was recently suggested to be involved in cell cycle arrest of MODY4 cells (40). Since *TP53* mutations probably provide the cells with selective advantages and since these mutations remain and expand in the culture during pancreatic differentiation (57), they could affect the phenotypes observed in the models they are derived from and their interpretation. These mutations should thus be tightly monitored when using hPSC for disease modeling.

### Epigenetic Aberrations

Another type of aberrations affecting hPSCs is epigenetic aberrations, including DNA methylation alterations, loss of parental imprinting, and variation in X chromosome inactivation (58). Specific genomic regions are prone to acquire hypermethylation leading to gene silencing during *in vitro* culturing and during reprogramming. These may affect the differentiation capacity of the cells, as was shown for *TSPYL5*, which was recently found to cause aberrant differentiation of cells upon its silencing (59). Various methylation aberrations were shown to originate from the reprogramming process of iPSCs, altering several genes in different cell lines (60). These aberrations, however, were suggested to diminish over time and passaging (61). Other methylation aberrations, usually corelated with cancerous mutations, provide growth advantage and are selected for during culturing (58).

Parental imprinted genes are genes that are expressed from a single allele, while the other allele is methylated and silenced depending on the parental origin. Wang et al. (43) found a decrease in PDX1 targets upon its haploinsufficiency in pancreatic progenitors differentiated cells (43). Two of these genes were *NNAT* and *MEG3*, paternally and maternally imprinted genes, respectively, which are related to insulin synthesis and secretion. This finding is based on comparing isogenic control iPSCs and mutant generated *via* CRISPR editing (43). However, many of the MODY studies are based on mutant and control iPSCs from different genetic and epigenetic backgrounds, such as diseased and healthy family members. In these cases, imprinted genes may be influenced by loss of imprinting aberrations, that consequently cause an expression upregulation through the biallelic expression (62).

Another epigenetic aspect relates to MODY, and a diabetes model is the epigenetic memory affecting differentiation capacity. It was previously shown that hESC and iPSC can be differentiated towards *β*-like cells in similar efficiencies (21) and that the majority of differences between lines in that context were related to the genetic background of the hPSC donor (63). iPSCs derived from specific somatic cells were thought to differentiate better towards the same cell-type of origin. Specifically, iPSCs generated from human pancreatic *β* cells were shown to differentiate better than ESC or iPSC derived from fibroblasts towards pancreatic progenitor cells (64). It was proposed that this observation originates from the open chromatin sites in the *β* cell-derived iPSCs, that were found in genomic regulatory regions related to endoderm and pancreatic islets’ development, indicating an epigenetic memory of iPSCs (65, 66). This feature of iPSCs can be beneficial when modeling MODY, since *β* cells are usually formed and function in patients (6).

### Gene Editing

The high rates of point mutations and genetic aberration occurrence in hPSCs and their clonal mode of enrichment, together with differences in differentiation capacity between cell lines, highlight the need for carefully supervised controls. Many of the MODY studies discussed above used non-isogenic cells as control to the mutated cells, which may affect their conclusions. The introduction of gene editing and, specifically, the CRISPR/Cas9 methodology enables the formation of isogenic lines, with the same genetic background, enabling the study of the exact effect of a mutant gene. This can be done by either introducing a mutation to a healthy control background, or by correcting a mutation in iPSCs generated from patients. MODY models that utilized CRISPR editing used the former option, and moreover, created both monoallelic and biallelic mutations to study dosage dependence effect of the mutated gene (19, 36, 42).

Though the CRISPR system enables many promising applications (20), it has some limitations that should be considered. While the immune response and obstacles in vector delivery are the main pitfalls for its *in vivo* use in regenerative medicine, the off-target and low efficiency are the main disadvantages for *in vitro* disease modeling approaches (67). Off-targets are genomic regions that may be mutated, undesirably, when using Cas9, especially when it is constitutively expressed. It was also suggested that CRISPR editing in hPSCs is sub-optimal in the sense of efficiency, possibly due to TP53-mediated cell cycle arrest and apoptosis which are induced by the Cas9. These obstacles may be tackled by predicting the off-targets, restricting the Cas9 expression and using improved CRISPR protocols (67).

### *β* Cell Differentiation

MODY modeling requires the differentiation of mutant ESCs or iPSCs towards pancreatic cells. As mentioned above, differentiation protocols are diverse and the differentiation capacity varies between cell lines because of genetic and epigenetic background. Since different studies are based on different differentiation protocols, and use different types of cells (and in the case of iPSCs—different reprogramming protocols), the final conclusions may be affected and even cause discrepancies between studies (28, 29). Current protocols generate *β*-like cells, which are not fully mature *in vitro*, thus limiting the study of MODY mutations’ effect on the final stages of maturation and function of mature *β* cells. During recent years, several groups generated hESCs and iPSCs derived islet-like organoid. Such organoids containing MODY mutations are yet to be done. They could contribute to the study of these mutations’ effect on pancreatic cells other than *β* cells, for example the impact of *HNF1A* mutation on *α* cells (19) or even on the development of the islet as a whole (13, 26, 45, 49).

## Conclusions

In this review, we discussed the great power, as well as the limitations, of using hPSCs for modeling the monogenic diabetes MODY group of disorders. MODY are relatively rare diseases, underdiagnosed with multiple existing subtypes—modeling all MODY types is an ongoing quest. Indeed, nowadays, hPSCs models were generated only for a few MODY types (**Table 1**). Models generated so far contributed to our knowledge of essential pancreatic TF mode of action, the relation between them, and the mechanisms that cause the disease when those are mutated. These models are essential to studying human pancreatic development.

The field of pancreatic developmental disease modeling in hPSCs is still facing challenges, including the requirement for an improved robust and unified differentiation protocol for the generation of mature functional *β*-like cells *in vitro*. Modeling disease using hPSCs requires tight monitoring of genetic and epigenetic aberrations that may be acquired during reprogramming, culturing, and gene editing (**Figure 1**). However, the great improvement of gene editing technology as well as the increase in clinical identification of MODY patients will further promote MODY research thus, helping better understanding of human pancreatic development and offering new treatment options for patients.

## Author Contributions

CB-G wrote the manuscript with support and supervision from NB. All authors contributed to the article and approved the submitted version.

## Funding

This work was partially supported by the Israel Science Foundation (grant 494/17), by the Rosetrees Trust, and by the Azrieli Foundation. NB is the Herbert Cohn Chair in Cancer Research.

## Conflict of Interest

The authors declare that the research was conducted in the absence of any commercial or financial relationships that could be construed as a potential conflict of interest.
